# Morphometric Analysis of One-Component Polyurethane Foams Applicable in the Building Sector via X-ray Computed Microtomography

**DOI:** 10.3390/ma11091717

**Published:** 2018-09-13

**Authors:** Aurelia Blazejczyk

**Affiliations:** Department of Civil Engineering, Faculty of Civil and Environmental Engineering, Warsaw University of Life Sciences—SGGW, Nowoursynowska 159 ST., 02-776 Warsaw, Poland; aurelia_blazejczyk@sggw.pl or pub.wbis.sggw@gmail.com; Tel.: +48-22-5931000; Fax: +48-22-5931087

**Keywords:** X-ray microtomography, one-component polyurethane expanding foams, blowing gas, strut, macro-pore, volumetric distribution, foam morphology, cell topology

## Abstract

A detailed morphometric analysis of one-component polyurethane (PU) expanding foams, with densities of 26 and 28 kg/m^3^ (‘SUMMER’ and ‘WINTER’ product versions), was conducted to evaluate the topology of the foam cells and to discover processing-to-structure relationships. The microstructural analysis of the heterogeneously distributed pores revealed tight relationships between the foam morphology and the cell topology, depending on the growth rate and local environmental conditions, governed by the properties of the blowing gas used. The most significant morphometric output included the following: open/closed porosity and (heterogeneous) pore distribution, relative density and (homogeneous) strut distribution, and total solid matrix surface and closed pore surface area—at the macroscopic level of the foam. While, at the microscopic level of the cells, the results embraced the following: the size of every detected strut and pore, identified two-dimensional (2D) shapes of the cell faces, and proposed three-dimensional (3D) topologies modelling the PU foam cells. The foam microstructure could be then related with macroscopic features, significant in building applications. Our protocol outlines the common procedures that are currently used for the sample preparation, X-ray scanning, 3D image reconstruction and dataset analysis in the frame of the X-ray computed microtomography (µ-CT) testing of the one-component PU foams, followed by a statistical (multiple Gaussian) analysis and conceptual considerations of the results in comparison with thematic literature.

## 1. Introduction

Since being discovered in 1928, the development of technologies based on polyurethane (PU) continues to this days, while the global production of the PUs (of any form) exceeds 20 million tons. It is a huge industry and an important branch of the economy. Its value is in the order of 50 billion U.S. dollars (data for 2017 from the Statista portal).

The large-scale production of the flexible and hard construction PU foams, applicable in the building sector, started in the 1960s, 20 years after Otto Bayer successfully synthesized PU foam. The flexible foams (one- and two-component foams [1]) were distributed either as panels or in cans. The former have been applied in building as acoustic insulation, and the latter as fillings of cracks in joinery; sealing and noise nuisance in windows, shutters, stairs, cable or pipe conduits; and water-sanitation or plumbing, as well as insulation in central heating or cooling systems. The hard construction foams could be spray foams discharged from a barrel to serve as thermal insulation (e.g., of attics); or, they could be panels for the thermal insulation of house walls.

Research of the PU foams has been underway for 80 years. It was recognised that the microstructure governs the physical macroscopic properties of the material (thermal, acoustic, strength, and other mechanical, etc.). However, the most challenging community aim has been to relate the macroscopic properties with microscopic features. The former may also include the foam density, relative density, or volume contribution of the solid matrix, its surface, or the total pore area and the foam open/closed porosity or relative pore volume, as well as the statistical distributions of the pores and struts—determining the PU foam morphology. The latter may be polyhedral topology and the size of a foam building cell (pore), with its polygonal walls or windows (faces) with struts (edges) and their triple- or quadruple-strut nodes (vertices). Also, the topology state of openness/closedness of the cell and the way the nearest cells are packed, thanks to which the foam is formed. Therefore, the intention of this study is to contribute to community efforts—to relate the PU foam microstructure with macroscopic features—by evaluating topology at the cell level, while measuring the morphometric parameters of the structure (surface and volume, relative density, porosity, etc.) and exploring the topological homogeneity based on a statistical analysis of the pore and strut size distributions at the foam level.

Herein, the term ‘morphometry’ is used in the sense of a quantitative analysis of a solid two-dimensional (2D) shape or three-dimensional (3D) form, a concept that encompasses the size measurement, topology evaluation, morphology outline, and visualisation. In this study, the geometric morphometrics were used to quantify a trait of evolutionary significance, by detecting changes in the foam morphology and topology of the microstructural elements. A major objective of this concept was statistically testing a hypothesis about the factors that may affect morphology, and discovering the structural evolution induced by the changing factors (such as blowing gas). These were determined by processing two different foam formulations. The structural characteristics versus the microstructure have been researched extensively for polymer foams [1,2,3,4,5,6], ceramic foams [7,8,9,10], metal foams [9,11,12,13,14], and carbon foams [15,16,17,18,19], yet, there is no in vitro evidence concerning the relationship between the structural characteristics and microstructure optimisation upon expanding of the one-component PU foams applicable in the building sector, based on the scaffolds of different morphology.

Among several standard techniques (see Table 1), X-ray computed microtomography (µ-CT) can be used as a special morphometric tool for measuring and visualizing of the 3D microstructure of solid materials, specifically of porous cellular [20,21,22]. In general, pores in solids are classified as shown in Table 1 [23,24]. The resulting digitalized 3D model of the cellular porous material may also contain location of the pores, struts, nodes, and walls (coordinates).

In this study, the X-ray scanning of the PU foam samples could be followed by an automated 3D image analysis to extract the structural information about the pore and strut form, number, and size. The term ‘pore’ could be geometrically defined for solid foams in different ways (see below), however, in this analysis, it refers to the void space, which is embraced by the cell walls or the cell windows (if eyelets exist). In this context, the pore size can be estimated as the diameter of the largest sphere inscribed (effectively fitted) into the limited pore space, formed by a ‘bubble’ polygon cluster. The term ‘strut’ refers to a of matrix skeletal frame ligaments that includes the nodes at both its ends. In this context, the strut size can be estimated as the diameter of the largest sphere inscribed and limited by the surface of such strut solid [25].

Considering pore size defined by the International Union of Pure and Applied Chemistry (IUPAC), the pores can be micro- (even though they have nano-metric size), meso-, or macro-pores [26,27,28]. The micro-pores form an adsorption area for gas molecules; while the meso-pores form the area of capillary condensation and allow for gas diffusion; and the macro-pores form the space for laminar fluid flow (gas or liquid) and, most of all, influence bulk density. The real macro-pores (up to 100 µm) and the over capillary macro-pores (up to 1000 µm) could be detected in the PU foams under study. The terms ‘nano-pore’ or ‘nano-porous materials’ can also be used. The terms ‘nanofoams’, ‘nanocellular’, or ‘nanoporous’ may refer to thermoplastic foams, which are a novel class of material with a cell size in the order of 10 nm [29]. In the case of carbon materials, the definition of nano-pores has not been established yet [24].

Considering the pore topological state (openness/closedness), the pores can be open or closed. Additionally, there are partially open pores and mixed pores in a foam microstructure [30,31]. Usually, open pores can be detected by the GA (gas adsorption) technique [26,27,28]. However, in the case that the pores are too small for the testing gas molecules, they can be detected by SEM (scanning electron microscopy) measurements or SAXS (small-angle X-ray scattering) measurements, which also identifies whether they are either open or closed pores [24,32,33].

Considering the strength and thermal behaviour, the cells can be rigid or flexible. Because of the different matrix strength, the hard cells are durable and high-temperature-proof, while the soft cells are easily deformable and could break under external stress.

Considering the origin of the pore formation, the intra-pores exist inside, while the inter-pores exist amongst the individual particles, crystallites, grains, or cells. The intrinsic intra-particle pores originate from imperfections of the crystal structure itself (e.g., interlayer space between hexagonal carbon layers stacked in carbon materials). The extrinsic intra-particle pores can be induced by doping a foreign substance (e.g., narrow pores between the expanded graphite layers by the intercalation of ions or particles of potassium, boron, nitrogen, or more a complex compound) [24,34]. The inter-particle pores may be formed by the space between molecules or chains (e.g., in polymers) [35,36,37]. Intra-grain pores could be inside a bead of expanded polystyrene (EPS), expanded polyethylene (EPE), expanded polypropylene (EPP), or thermoplastic polyurethanes (TPUs), while the inter-grain pore is the void of space among the beads, in the case of granular foams [38,39,40]. The intra-cell pore is the void space limited by the walls within the closed cell, for example, extruded polystyrene (XPS), while the inter-cell pore is void space limited by walls of neighboring foam cells or an intersection of two open cells, for example, Duocel^®^ open cell foams (from ERG Aerospace, Oakland, CA, USA), in the case of non-granular foams [41,42,43,44,45,46].

## 2. Materials and Methods

### 2.1. Investigated Material

The investigated PU mounting and sealing foam is an expanding foam, also known as ‘one-component foam’ (OCF). It was manufactured by Soudal Sp. z o.o. in Poland (Soudal N.V. Belgium subsidiary, Turnhout, Belgium). The material, wholesaled in aluminum cans, is commercially available in the two product versions, ‘WINTER’ (W) and ‘SUMMER’ (S). While the S-foam version is for use within the temperature range of 10 to 30 °C, the W-foam is designed for application from −10 °C upwards.

A single dispenser (can closed with valve) with the OCF product contains the following:

- liquid prepolymer functionalized with isocyanate groups (pre-reacted from a mixture of polyol and polyisocyanate),
- additives (flame retarder and chemical stabilizer of prepolymer),
- physical blowing agents (mixture of dispersed and partially dissolved in prepolymer compressed-liquefied inert gases),
- vapours of gas mixture in an ‘empty’ part of the can (remaining in thermodynamic equilibrium at the gas–liquid interphase, under pressure of 2–4 bars).

Both of the product versions are based on the same prepolymer, yet they differ in their blowing gas mixture composition. Some information about gases, including the gas mixture components and their percent mass range, could be found in a safety data sheet (SDS), provided by the manufacturer. The information is shown in Table 2, together with some selected physical properties taken from the National Institute of Standards and Technology (NIST). Each inert gas component plays the role of both a propellant and blowing agent.

### 2.2. Sample Preparation

In each case, the dispenser content (with W- or S-foam) was injected into the casting mold, and moisturized with water sprayed at 20 °C. This allowed for free expansion and receiving the cuboidal PU panels, which were later stabilized for two weeks, under quite stable laboratory conditions—at 20 ± 1 °C, 50% ± 5 % RH, and 1000 ± 10 hPa.

**Figure 1:**
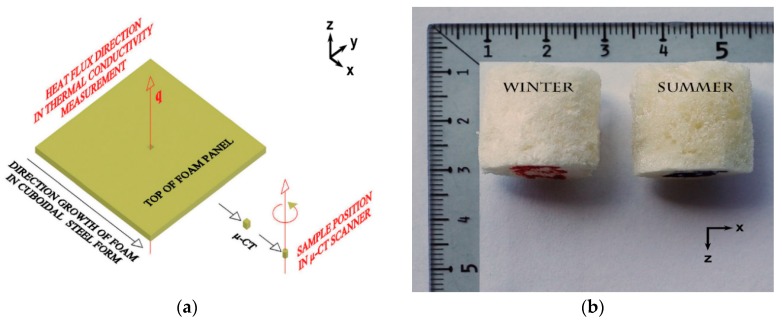
(**a**) Preparation of the sample for X-ray computed microtomography (µ-CT) measurement from the polyurethane (PU) panel: foam growth along the x-axis, cutting a cuboidal, and further forming the cylindrical sample. The sample rotation in the µ-CT scanner is shown around the z-axis—parallel to vector q and perpendicular to the scanner holder in xy-plane. In this figure, the sample to panel size ratio is kept the same as the original; (**b**) The enlarged WINTER (W)/SUMMER (S) foam samples are shown over the xz-plane.

From each hardened PU panel, about half way from the center to the edge, a small ‘cube’ was cut out, from which the cylinder was next formed—as shown in Figure 1a,b.

The cylindrical form was selected on purpose, as it is recommended in the µ-CT practice to scan the rotational solids of the symmetry axis parallel to rotation axis (z) of the scanner holder (on xy-plane). The X-rayed sample of the cylindrical form, during rotation in the chamber, throws the shadow (rotation image 2D vertical projection, scan) of the constant area, independent of its angular position. This facilitates the sample microstructure reconstruction in further data processing. The cylindrical samples were cut out from the panels along with the direction of the foam growth (x-axis). This is important when considering microstructure anisotropy. Some microstructure anisotropy (in terms of cells expansion towards x-axis) is expected. This effect might cause the directional dependence of the thermal conduction in the hardened PU foam panel. After the scanning process, the samples were stored and will be used in further research, with a major consideration of anisotropy.

Next, both the prepared W- and S-foam samples were stacked and fixed by a special glue layer. Then, the stack was glued with the scanner holder before the X-ray µ-CT measurement. The glue layers were later digitally cut out before reconstruction, to avoid affecting a further microstructural analysis.

Some specifications, concerning both of the tested PU foams, are listed in Table 3.

The bulk density, *ρ*, was determined for each sample through measuring the mass (with Sartorius CP 324 S-OCE balance), the cylinder dimensions (by using digital caliper), and calculating the mass to volume ratio (with estimated uncertainty ±2 kg/m^3^). The measured density resulted somewhat higher than what was declared by the manufacturer—as a result of the constraints imposed on making each PU panel, which grew in the casting mold that was closed with a cover, which caused a slightly increased pressure (relative to atmospheric) during such hindered expansion. Moreover, the breakage of pores occurs at the surface when the cutting and shaping the samples into the cylinder form (caused by using a precise stainless-steel tool). This effect does not change the sample mass but it may slightly reduce the volume, so the density (measured as mass to true sample volume ratio) increases.

### 2.3. Experimental Setup

Scanning of the prepared samples was done using the Skyscan 1172 scanner (Bruker microCT, Kontich, Belgium), an X-ray microtomographic system (by Bruker microCT, Kontich, Belgium). The main technical specifications of the microtomograph are given in Table 4.

### 2.4. Scanning Procedure

The µ-CT scan settings used depends on the samples’ physical properties, and aims to achieve the best image quality [25,47,48,49,50,51,52,53,54,55,56,57,58]. In the case of the tested PU foam, the cylindrically shaped samples were visibly porous, with mostly 2–3 mm blow holes, and extended blisters clearly appeared on the exterior curved surface. To visualize the foam structure, which, besides all of the others is formed from such large pores, the sample should be at least one order of magnitude larger. Also, the smaller the sample size (cross section width or height, whichever larger), the higher magnitude and the resolution achievable with the scanner, as a consequence of the SkyScan 1172 adaptive geometry. Hence, the sample size (Table 3) and the resolution had to be optimized, together with scanning time and other scanner settings (Table 5).

The scanning datasets for the W- and S-foam samples were obtained in the cone X-ray beam acquisition. The acquired dataset consists of 2925 (195 ÷ 0.2 × 3 = 2925) scans in a 16-bit TIFF (Tagged Image File Format) format (4000 × 2664 pixels) for the two sample stacks. The total X-ray exposure time (1680 ms) includes the number of the four frames, which were averaged at each angular position of the scanner.

The scanning process, carried out at a constant temperature, took about 3 h. As the PU foams are known to have a good dimensional stability in a wide temperature range [59], one may assume that the chamber temperature variation of ±5 °C did not affect the material structure parameters during the scanning process.

### 2.5. The 3D Image Reconstruction from the Scans

For each prepared sample, the 3D cross-sectional image stack (3D stack) was reconstructed from the acquired scans, using the NRecon X-ray cone beam reconstruction software (by Bruker microCT, version: 1.6.9.8, Kontich, Belgium) based on the Feldkamp algorithm [60]. The NRecon software allowed for adjusting four initial reconstruction parameters: smoothing, misalignment compensation, ring artifact reduction, and beam-hardening correction. All of the parameters had to be found experimentally via the trial and error method, and with reference to the literature [61]. The resulting optimal settings are listed in Table 6.

Before launching the reconstruction, the appropriate region of interest (ROI) was selected. The ROI is defined as a locally selectable region in a single 2D cross-sectional image. Its reference square size is 4000 × 4000 pixels by default. The new ROI size had to be reduced in order to crop the excess background and reduce the file size in bytes, and its choice was realized after viewing a few 2D cross-sectional trial images displayed in the NRecon preview mode—selected along the 3D cross-sectional image stack. Each of the sample cylinder 2D cross-sections had to be well inscribed in the selected ROI square. The optimal selected ROI was assigned to all of the 2D cross-sectional images to be reconstructed.

Then, the 3D image reconstruction was performed within the volume of interest (VOI). The VOI refers to a 3D cross-sectional image stack made of a given number of the ROIs, which were integrated according to the Feldkamp algorithm. The VOI size was defined by the setting position (nr) of both the first and last section. The choice of the VOI is based on careful consideration of the PU foam structure, which revealed an uneven distribution. The VOI height could not be too short, otherwise some interesting morphology changes or macroscopic structural effects could not be detected. On the other hand, the VOI also could not be too tall because of computing constraints. Additionally, to avoid affecting the microstructural analysis of the foam samples, all of the glue layer images had to be digitally removed. In effect, the reconstruction started by setting the first and the last section positions, as in Table 6. The last sections were set at each sample’s mid-height (11.1 mm). Consequently, the selected VOI (cuboid) corresponded to more than half of the initial sample volume, for both the W- and S-foam samples. This saved the computing time without affecting the resolution and precision during further analysis. The selected VOI size was large enough to be representative for material, and to determine all of the structural traits of both PU foams.

Hence, the resultant reconstructed dataset consisted of 1771 2D cross-sectional images in a 8-bit BMP (Bit Map) file format—for each W- and S-foam sample. The 16-bit TIFF format, despite containing more information than the 8-bit BMP, does not allow for saving a colour palette, and allows objects to be represented in greyscale only. Colours may be of importance for a further detailed image analysis (in the CTAn software, version: 1.13.2.1, Bruker microCT, Kontich, Belgium) and visualisation (in the CT-voxel software, version: 2.4.0.0, Bruker microCT, Kontich, Belgium).

### 2.6. Automated 3D Image Analysis

The obtained 3D cross-sectional image stacks (saved by NRecon as two sets of the 8-bit BMP files) were further automatically processed and analyzed by the CTAn software (by Bruker microCT) [62]—for both the W- and S-foam samples—to extract structural information.

**Figure 2:**
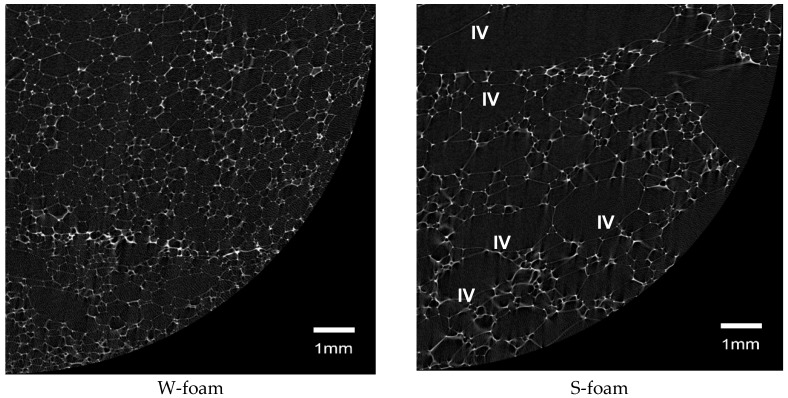
The example of horizontal (xy-plane) two-dimensional (2D) cross-sectional quarters of the reconstructed 3D cross-sectional image stacks, representing the PU W- and S-foam (BMP not resized, image pixel size 6.25 µm). The 2D cross-sections are the last 3D stack sections, numbers 2100 and 5420, respectively. The white pixels in the reign of interest (ROI) represent the PU foam struts, the dark grey pixels—void space and the black—background. The symbol ‘IV’ denotes the population of largest pores, visible examples (cross-section) even with the naked eye in this image.

Before the analysis, which was carried out for each sample after loading 1771 of the BMP images, the ROIs had to be re-selected, in order to consider identical sample regions and to avoid accounting for irregular border effects. The new ROI was selected by setting its new shape (circular), its new size (Ø 18 mm), and location in each of the 2D cross-sectional images, corresponding to each 3D stack. Examples of the 2D cross-sectional images in their re-selected circular ROIs are presented in Figure 2, for both the W- and S-foam samples (only quarters).

Consequently, the new VOI was re-calculated for both 3D stacks. Next, a 3D image analysis and visualization were performed within the updated VOI.

Then, the 2D cross-sectional images in greyscale were binarized by setting a precisely selected lower grey-level threshold, above which the pixels were considered as material and below which they were considered as background. The thresholding (segmentation) was done on a histogram from the dataset for both of the entire 3D stacks, using the simple global method.

The last tasks before the analysis were to realize the ‘Despeckle’ and ‘ROI shrink-wrap’ procedures. White speckles had to be removed from the 3D space of the stack image, wherever the speckle volume was less than the given number of voxels. Also, the ROI could be shrunk to the defined boundary of the binarised 2D cross-sectional image, by stretching the black background over the blowholes (cross-sections of the outermost cells) of the diameter above a given number of pixels. Hence, this number relates to the microstructure of a given sample material. The bigger the blow holes, the higher the stretching level. Consequently, the caves with an opening larger than the given diameter were removed from both of the 3D stacks.

The optimal values of the key input parameters and settings for the analysis are shown in Table 7. The outputs of the analysis, the two sets of morphometric parameters describing two different microstructures of the PU foam under study, are shown in Table 7.

To provide a reason for the resulting difference between the levels of 44 and 58 stretching over the blow holes, one may notice that the effective cell size is apparently increased (comparing S- to W-foam examples in Figure 2). Then, in order to keep approximately the same shrunken ROI area, the stretching had to be set on an upper level.

## 3. Results

### 3.1. The CTAn Output and Statistical Analysis

In this section, the main morphometric parameters are presented, describing the structure of both the W- and S-foam samples [63]. The output parameters, resulted after the CTAn analysis, are presented in Table 8. It is worth highlighting that the term ‘pore’, used by CTAn software, refers to the combined void space embraced by the ‘cell’ and its ‘windows’. Thus, the pore size was calculated by measuring the diameter of the maximal sphere inscribed into every detected cell and its windows space. 

#### 3.1.1. Parameters and Relations

The following expressions explain the relations between the parameters listed in Table 8:

TV = Obj.V + Po.V(cl) + Po.V(op)(1)

Po.V(tot) = Po.V(cl) + Po.V(op) = TV − Obj.V(2)

Po(cl) = Po.V(cl)/Obj.V × 100%(3)

Po(op) = Po.V(op)/TV × 100%(4)

Po(tot) = (Po.V(op) + Po.V(cl))/TV × 100%(5)

*ρ*_r_ = *ρ*/*ρ*_s_ = (*m*_b_/*V*_b_)/(*m*_s_/*V*_s_) ≈ *V*_s_/*V* = Obj.V/TV (6)

Po(tot) = Po.V(tot)/TV = (TV − Obj.V)/TV ≈ 1 − *ρ*_r_ (7)

where *m*_b_ and *V*_b_ are the sample bulk mass and volume, respectively, *m*_s_ and vs. are the solid matrix (no gas) mass and volume, respectively, *ρ* is the foam bulk (both solid and gas state) density, *ρ*_s_ is the matrix density (only solid state), and *ρ*_r_ is the foam relative density (contribution of the solid matrix to the sample volume) [31].

One may notice from Equations (3)–(5) that both the open porosity Po(op) and total porosity Po(tot) are expressed as a percentage of the total volume (TV), yet, the closed porosity (Po(cl)) is expressed as a percentage of the matrix volume (Obj.V) (in fact, it is only matrix that forms the closed pores rather than much larger total void space in the sample, so the contribution of the closed pore volume refers to a fraction of the matrix rather than to the total sample volume). Thus, Po(tot) is not the sum of both Po(cl) and Po(op). As results from Equations (6) and (7) show, both the parameters, Po(tot), and the relative density, *ρ*_r_, add up to unity. Moreover, the Obj.V/TV ratio has the physical sense of relative density, *ρ*_r_, which is a measure of the solid matrix contribution to the total sample volume. As seen from Table 8, the *ρ*_r_ ≈ Obj.V/TV parameter results were higher for W-foam, so, as expected, the more volume content of solid matrix Obj.V, the more volume of the closed pores Po.V(cl), and the higher their surface Po.S(cl).

As seen from Table 8, the total porosity results were greater by approximately 2% (that is [90.252 − 88.440]/88.440 × 100%) for S- than for W-foam. The same percentage change is evident for the open porosity, although the closed porosity resulted in about a 56% (that is [0.004 − 0.009]/0.009 × 100%) lower for the S-foam, with respect to the W-foam. It indicates that the cellular structure is more open in the case of S-foam. Also, the total area of matrix in VOI to the total area of cylindrical VOI surface (Obj.S/TS) ratio resulted in about 31 for the W-foam sample and 25 for the S-foam sample. This result indicates that the complex structure of the solid matrix is more tightly and more densely packed in the 3D space of the W-foam relative to the S-foam.

#### 3.1.2. Pore Distribution

Beside the morphometric parameters, the CTAn output data also includes the volumetric distribution of the pore size (‘structure separation’) and strut size (‘structure thickness’) for both the W- and S-foam. In addition, the number distribution of the pore size could be directly derived from the volumetric distribution, according to the following formula:

(8) $$\frac{N\left(d_{i}\right)}{N_{\mathrm{total}}}=\frac{6\;V\left(d_{i}\right)}{\pi \;d_{i}{}^{3}\;\sum_{i}N(d_{i})}$$

where *N*_total_ is the total number of pores in VOI, *N*(*d*_i_) is a number of pores of a given size *d*_i_, and *V*(*d*_i_) is the volume of the pore population, indexed by i = I, II, ..., and so on.

**Figure 3:**
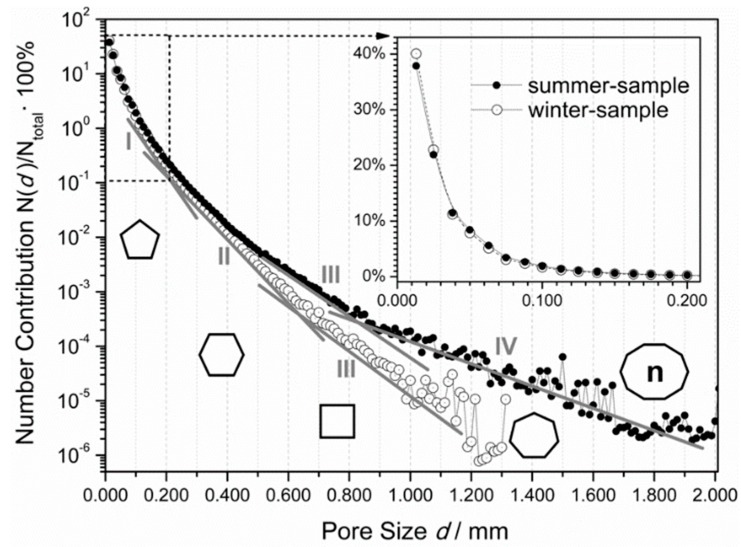
The number distribution of the pore size for both the W- and S-foam. The number contribution *N*(*d*)/*N_total_* of the pores of a given size to the total pore number in volume of interest (VOI) (BMP not resized, image pixel size 6.25 µm), shown as a function of the pore diameter, *d,* and plotted in the half-logarithmic scale. The inset plotted in the linear scale is to enlarge the highlighted 0–200 microns range. The solid lines, numbers I, II, III, and IV are guiding the eye along the apparent straight intervals of a gradually variable slope. The shapes of the cell faces in the growing pores (5-, 6-, 4-, and 7-sided, with the last n-sided polygon, where 7 < *n* < 20) are assigned to the corresponding intervals (I–IV), also given their frequency of appearance in the expanding PU foams (see discussion).

**Figure 4:**
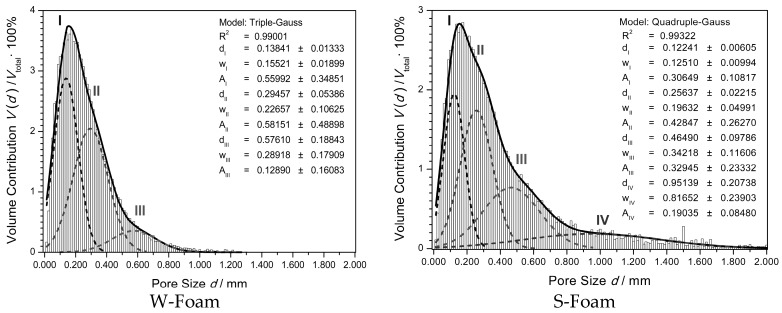
The volumetric pore size distribution for both the W- and S-foam. The volume contribution *V*(*d*)/*V*_total_ of the pores of a given size to the VOI (BMP not resized, image pixel size 6.25 µm). Each bar graph shows the apparent pore size distribution, the overlaid thick solid line represents the sum of the overlapped peak-components (I–IV), and the dashed lines represent the distributions separated by Gaussian analysis.

The resulted numerical and volumetric pore size distributions are presented in Figure 3 and Figure 4. Each volumetric distribution of the pore size was further processed in a statistical (Gaussian) analysis—to be discussed in Section 4.

The volumetric distribution, shown in Figure 4, describes the volume contribution *V*(*d*)/*V*_total_ of the pores of a given diameter (*d*) to the total VOI space, either for the W- or S-foam. The explicit maximum of the fitted solid line (distribution mode) results for both were 0.155 ± 5 mm and reached 3.8% for the W-foam and 2.9% for the S-foam. Its position could be compared with the mean pore size values −0.271 mm and 0.418 mm (‘structure separation’ in Table 8). The distribution spread, standard deviation (SD), is 0.182 mm and 0.398 mm for the W- and S-foam, respectively.

To account for the apparent distribution, the Gaussian model, a combination of the normal distributions, was fitted to each set of the raw data. The results showed that the apparent distribution could be well approximated by using the following general formula:

(9) $$\frac{V\left(d\right)}{V_{\mathrm{total}}}=\overset{j}{\underset{i=1}{\sum }}\frac{A_{i}}{w_{i}\sqrt{\pi /2}}\;e^{\frac{-2{\left(d-d_{i}\right)}^{2}}{w_{i}^{2}}}\;$$

The parameters of the fitted Gaussian model (9), *A*_i_, *d*_i_, and *w*_i_—denoting the distribution peak area, peak center, and its width, are shown in Figure 4, with their resulted values. For the W-foam, j = III, and for the S-foam, j = IV.

The i-number of a given Gaussian peak corresponds to the consecutive number of the *N*(*d*)/*N*_total_ function slope change in Figure 3, which is due to the existing relation, derived from Equation (8), as follows:

(10) $$\;\log\frac{V\left(d\right)}{V_{\mathrm{total}}}=\log\frac{N\left(d\right)}{N_{\mathrm{total}}}+3\log\left(d\right)+C\;$$

where C = log(π*N*_total_/6*V*_total_)—so that each new apparent gradient on the numerical distribution function indicates on a new possible Gaussian peak in the volumetric distribution at the corresponding pore size interval.

As seen in Figure 4, the overlapped distributions (labeled by I, II, and III, for the W-foam, and I, II, III, and IV for the S-foam) could be effectively separated by the Gaussian analysis and overlaid in the same graph. The heights of the component peaks (maximum percent contribution) decrease with the increasing pore size, *d*, from 2.9% down to 0.4% (W-foam), and from 2.0% down to 0.2% (S-foam), so the population of the smallest pores has the highest numerical and volumetric contribution in both the W- and S-foam microstructure.

Interestingly, unlike the W-foam, the S-foam reveals a more complex microstructure, due to the apparent (fourth) component of the largest pores distribution. This makes the S-foam distribution spread wider (compared to the standard deviations, ca. 0.2 and 0.4).

#### 3.1.3. Strut Distribution

**Figure 5:**
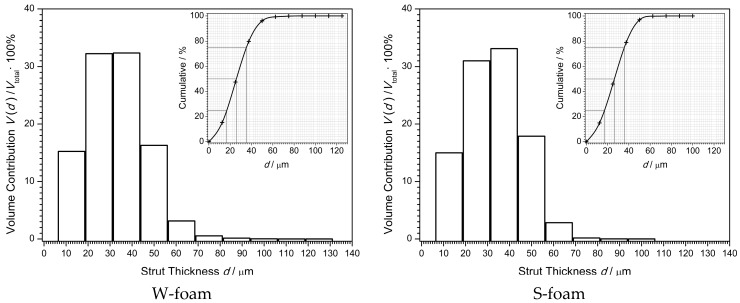
The volumetric strut (thickness) distribution for both the W- and S-foam (BMP not resized, image pixel size 6.25 µm).

The volumetric distribution, shown in Figure 5, describes the volume contribution *V*(*d*)/*V*_total_ of the struts of a given diameter, *d*, to the total matrix volume (Obj.V), either for the W- or S-foam. Each distribution is slightly skewed right, with a longer right tail, left-modal, yet, it could be well approximated by standard normal distribution.

To account for each apparent distribution, the cumulative distribution was plotted and its basic parameters were found, such as the mean, SD (standard deviation), median, IQR (interquartile range), and mode.

Interestingly, the W-foam and S-foam reveal approximately the same distribution of the structure thickness—due to the fact that, unlike pores, the strut (thickness) distribution parameters results were almost exactly the same. Similarly, the strut thicknesses extend in the narrow range, roughly from 10 to 100 µm. In agreement with Table 8 (see ‘structure thickness’), the mean values resulted both 33 µm (with SD 13 µm), median values 26 and 27 µm (with IQR 18 and 19 µm—as a measure of spread), and mode from 31 to 44 µm (the most frequent strut thickness).

### 3.2. The Structural 3D Visualization

**Figure 6:**
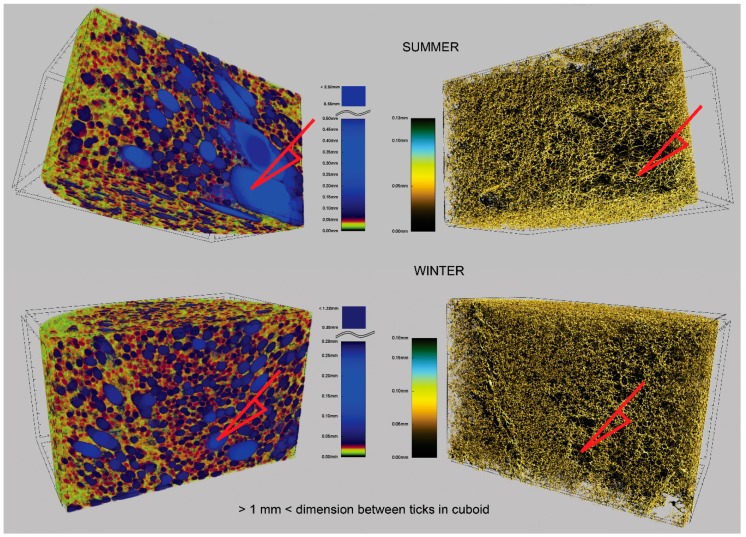
The S- and W-foams 3D realistic visualization screens (image pixel size 25 µm)—mapping the pore system (left) and matrix skeletal frame (right). The scales assign colors to the given pore size and strut thickness. The red arrows point corresponding regions on the pore map and the strut map; they are also to show visible difference in pore size comparing the W- with S-foam (SUMMER/WINTER).

The 3D image analysis (described in Section 2.6 and Section 3.1) was completed using CTvox (CT-voxel software by Bruker microCT) [64]. The CTAn output (3D cross-sectional image stack) was loaded by CTvox and transformed into the realistic structural 3D visualisation for each virtual sample. It allowed for viewing by rotating any part of the skeletal frame (forming the solid matrix network) and the pores (forming the mostly open channels network). In Figure 6, there are examples of the screens displayed during visualizing the strut network (right hand side) and the pore system (left hand side), for both the W- and S-foam.

In Figure 6, each 3D box base is in the horizontal xy-plane, and the front wall is in the vertical xz-plane. The mixture of blowing gases escaped through yz-plane of the PU foam sample, towards the right hand side. The 3D box dimensions are about 9 mm (depth), 18 mm (width), and 11 mm (height), which correspond to approximately a quarter of the initial PU sample volume.

## 4. Discussion

During the foam expansion, a blowing gas filled pore increases its size under the pressure exerted by its local surrounding environment. The faster the foam becomes harder, the smaller the pore that can be formed. And the greater the vapour pressure of a given blowing gas, the larger the pore, together with its struts. Therefore, the factor that determines the eventual pore (and its struts) size is a competition between the blowing gas expansibility and the dynamics of the viscosity changes of the solidifying polymer matrix.

### 4.1. Pore Distribution

From one side, the exterior pressure experienced by the expanding bubbles (that eventually become cells or pores) may vary because of random fluctuations in their local environments, changing throughout the overall sample space. On the other hand, the interior pressure also may slightly vary, as foam expansion is initiated by absorbing single vaporized blowing gas molecules that diffuse to the growing bubble in a random way. Thus, the pore population should be governed by a specific statistical distribution of the pore size. The most natural distribution in the case of homogenously porous, yet polydisperse foam, that is, with lots of the cells of a similar 3D geometrical form, but of slightly varying volumes, would be the standard normal distribution (volumetric). Therefore, one could assume that a single Gauss distribution would properly describe the variability in the pore size, if all of pores had the same 3D form.

Interestingly, as seen from Figure 4, the pore distribution for the W-foam reveals a visible asymmetry, which is even worse for the S-foam—with the right distribution wing being much wider, exceeding 1 mm. In this case, a single Gaussian peak would not be enough to account for this effect. Consequently, it has been proposed and has succeeded to fit the ‘Multiple Gauss’ model to the apparent distribution. As seen, the computed resultant sum of the Gaussian components (I–II and I–III for W- and S-foam, respectively) seem to provide a good envelope (solid line) for each apparent distribution (each bar graph). As the ‘Triple-Gauss’ and ‘Quadruple-Gauss’ models closely approximate each resulted distribution, they could be used to analyze both foams. The apparent asymmetry may indicate that the foam material is heterogeneously porous. In other words, either the W- or S-foam might comprise at least three coexisting types of the macro-pore microstructures, which base on the unit cells, revealing at least three different topologies of their 3D forms.

The macro-pores reveal diversity in cell forms, due to possibility of forming (by linking struts) more or less regular polyhedral forms [48,65,66,67,68], and their irregular randomly distorted combinations. As most often found in the studied foams, the complex architecture of the PU foam structure could be modeled by using the classic Kelvin (K), Matzke (M), or the most recent Weaire–Phelan (W–P) unit cells—described by the following features [69,70]:

- The K foam cell topology bases on the 6-0-8 14-hedron, including six quadrilaterals (no pentagons) and eight hexagons, which often might rearrange into the Williams cell—2-8-4 14-hedron (W). The energetic ‘cost’ [71] of constructing a single K cell, related to its surface free energy, is lower than W (*S·V*^−2/3^ = 5.336 dimensionless), yet, at least equals 5.315 or more (depending on cell anisotropy and surface tension).
- The M foam unit cell topology bases on the most abundant 1-10-2 13-hedron or on the much less frequent 1-10-3 or 3-7-3-1 14-hedra, including at least one quadrilateral and even one heptagonal face. The ‘cost’ of the M cells starts from 5.314 (1-10-2), which is better than the K cell.
- The W–P foam duplex unit cell is combined from the eight building blocks of the two topologies, the irregular 0-12-0 dodecahedron and the 0-12-2 tetrakaidecahedron, including 12 penta- and 2 hexagonal faces. The ‘cost’ of constructing the W–P duplex space tiling is ca. 5.288, which is much better than the others, yet, the ‘cost’ may still drop down to the minimum of 5.256 for 0-12-0.

**Figure 7:**
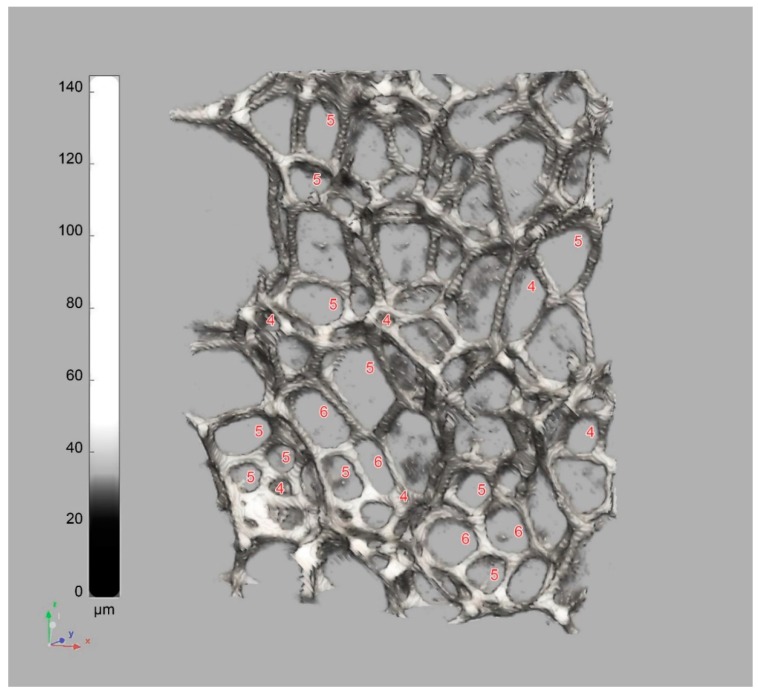
The PU foam microstructure, a cutout from the reconstructed W-foam sample (µ-CT resolution 6.25 µm). The cell faces are labeled with numbers of their edges. The greyscale corresponds to strut thickness.

As reported in literature, in PU foams, the most frequent 2D shapes of the cell faces, reveal a pentagonal geometry (up to 70%); while the hexagonal shape frequency exceeds 20% and the quadrilaterals less than 10% [48,70,72]. As also found in this study, the PU foam microstructure exhibits the cell faces mostly of quite regular penta- or hexa-gonal shapes, and rarely of the quadrilateral or heptagonal shape (see the cell windows in Figure 6 and Figure 7). However, the additional of more complex 2D shapes also exist and often appear, especially in S-foam (see Figure 2 and Figure 3).

Therefore, the 3D form of the PU foam polyhedral cells, shaped from a group of such faces, could be basically described by both the duplex W–P and M topology, and could be conjectured from their disordered clustered combinations. However, as the foam microstructure evolves toward a local minimum in the surface free energy, some topological transitions may occur as a result of initial stress relaxation during expansion and further structure stabilization. As described by Kraynik et al. [69], the Matzke cell 1-10-2 13-hedron can easily reduce its surface free energy by losing the quadrilateral face and rearranging into the smaller 0-12-0 12-hedron through the reverse topology change.

That may considerably reduce the M cell population and relatively increase the dodecahedra population in the PU foam. Hence, the smaller pentagon based 12-hedra (0-12-0) volumetric distribution can even dominate over the larger hexagon based 14-hedra (0-12-2), which consequently dominates over the quadrilateral based polyhedra of the M topology (1-10-2).

Therefore, taking into consideration both the cell construction ‘cost’ and the frequency histogram of the cell face polygons, one may accomplish the pore distribution interpretation as follows:

- The first two Gaussian peaks (I and II) extracted from the volumetric distribution (Figure 4) could be interpreted as originating from the pore population of the irregular pentagonal 12-hedra and of the W-P 14-hedra, respectively. The modal values of the two dominating effective fitted distributions are the same (0.155 mm), which indicates on the smallest pore populations formation as dependent on the material features (polymer chains structure and interaction, prepolymer viscosity and surface tension in cells) rather than the blowing gas physical properties.
- The third Gaussian peak (III) extracted from the volumetric distribution could be interpreted as originating from the disordered clustered combinations of the M type pore cells. As seen from Table 8 (structure separation), the W-foam reveals effectively smaller mean pore size (0.27 mm) than that of the S-foam (0.42 mm). This effect is due to relative expansion of the third and appearance of the fourth pore distribution component (Figure 4).
- While the third pore population formation seems to be still dependent on the PU material features, the fourth pore (IV) population depends rather on the blowing gas properties. The highest impact on the effect may have the saturated vapour pressure (SVP), which, according to the Antoine equation, is correlated with boiling point temperature (BP). As seen from Table 2, the lower the BP (at constant pressure), the higher the SVP (at constant temperature), and the higher the fugacity of the volatile gas. As can be seen, the only qualitative difference between the S- and W-foam is presence of the fourth component in the blowing gas mixture. The SVP (840 kPa) of propane is considerably higher than that of the 1,1-difluoroethane (516 kPa). Due to the highest fugacity, propane is the most volatile gas in the mixture. By replacing the 1,1-difluoroethane with propane, the effective pressure in expanding bubbles of the S-foam could considerably increase. In order to estimate the maximum difference between the S- and W-foam initial pressures in bubbles, one may assume the extreme compositions in the range given by the manufacturer (1–10%)—as shown in Table 9. The pressure, which initializes a single bubble growth, can be assessed by assuming equal chance diffusing and vaporing of the uniformly dispersed gas molecules into the bubble. So, the quantitative composition of the gas mixture in the bubble tends to the same as in the whole can. Thus, the total effective pressure of gas mixture in bubble *p*_total_ (maximum after initial rise) could be estimated, as the weighted average of the saturated vapour pressures, from the following:
  (11) $$p_{\mathrm{total}}=\sum_{i=1\;}^{j}\frac{n_{i}}{n_{\mathrm{total}}}P_{i}=\sum_{i=1}^{j}x_{i}\frac{M_{\mathrm{total}}}{M_{i}}\;P_{i\;}$$
  where *n*_i_ is the number of moles of the ith gas component, *n*_total_ is the total number of moles of gas mixture, *P*_i_ is the saturated vapour pressure of the ith component (Table 2), *x*_i_ is the mass fraction of the ith gas component, *M*_i_ is the molar mass of the ith gas component (Table 2), *M*_total_ is the molar mass of the W- or S-foam gas mixture, and j = 3 or 4 for the W- and S-foam, respectively.

Substituting the values from Table 2 and Table 9 into Equation (11), one may find that the pressure could rise even of 2.4 Bar higher, comparing the S- to W-foam. Such relatively high pressure could cause a dramatic disruption of the cell walls, breaking the struts in the still fluent matrix of the S-foam. Therefore, the last Gaussian peak (IV) extracted from the volumetric distribution (Figure 4) could be interpreted as originating from the population of the torn apart and linked cells forming anisotropic blisters. These pores stretched along the direction of the gas escape during the foaming process and are clearly visible as the largest blue blobs (Figure 6 on the left hand side), and even more so in Figure 6, on the right hand side, one can see the regions to which the big red arrows point.

### 4.2. Strut Distribution

Beside the pores, the resolution limiting this morphometric analysis (6.25 µm) allowed for detecting the struts, which seem to dominate over other structural elements, such as the thin cell walls or smaller struts that might exist in the foam matrix, yielding only up to 5% in the matrix volume (using the 95% confidence level with the strut thickness 33 ± 2 × 13 µm and the confidence interval from 7 to 59 μm). So, even if the resolution was somehow improved to 1 µm, the thinner struts that could be detected would neither contribute to the matrix network nor form extra cells to be considered.

The volumetric distribution of the struts, identified by their thickness, was shown in Figure 5 for the W- and S-foam. As mentioned in Section 3.1, there is no reason to state that the strut distributions statistically differ, which is independent on the presence of the 1,1-difluoroethane in blowing gas mixture. This effect could be explained as the following. When forming the fourth distribution of the largest pores in the S-foam, the higher vapour pressure of propane rich mixture of the blowing gases may naturally cause strut breaking. However, after the break, the stumps of a broken strut are immediately absorbed by the closest nodes—due to the surface tension of the liquid foam-forming viscous material. So, due to viscoelastic effects, the broken struts disappear and do not alter the apparent distribution.

### 4.3. Other Structural Traits

As also shown in Table 8, both the PU foams reveal an open-celled structure, indicated by the open porosity of nearly 90%. The difference in the blowing gas vapour pressure could result only in ca. 2% open porosity reduction in the W- compared to S-foam. In parallel, the volumetric contribution of the PU matrix to the total volume of the sample resulted in being nearly 20% higher for the W-foam, with respect to the S-foam, which is indicated by the relative density altering. One may also notice that the higher the relative density (more content of solid matrix), the greater the contribution of the closed pores, of which the content in both foams is, however, insignificant.

## 5. Conclusions

The W-foam differs from the S-foam only with the blowing gas mixture, especially 1,1-difluoroethane. The change in the mixture composition allowed for the lower application temperature of the “WINTER” product version, but probably hardly improved its thermal insulation properties or ability to seal closed spaces. It is because of the partial replacement of propane with the less volatile 1,1-difluoroethane (of nearly 40% lower vapour pressure and 50% larger molar mass) may not be enough to reduce the open porosity of the PU foam. In both product versions, the relative density (contribution of the matrix to sample volume) decreases too rapidly during the foam expansion, which starts already inside the dispenser. So, in order to increase the contribution of the closed pores, the dynamics (rate) of the foam expansion should be slowed down, even before the prepolymer exits the dispenser.

The 1,1-difluoroethane blowing gas component was found as responsible for altering the following:

- The foams’ microstructure at the microscopic level of cell in terms of the following:
  ○ the mean pore size (measured with 6.25 µm resolution).
  ○ the way of cell-to-cell linking (by a given type of interfacing window, pentagonal, hexagonal, etc.).
  ○ the pore morphology (manifested through various cell forms and window shapes)
- The foams’ structure at the macroscopic level of foam in terms of the following:
  ○ open and closed porosity.
  ○ matrix volume and relative density.
  ○ matrix surface and total closed pore area.
  ○ tighter pore size distribution (yet, the effect of the blowing gas could not be visualized by comparing strut size distributions, since the broken struts might be immediately absorbed by their closest nodes during foam expansion).

The morphometric studies, based on X-ray µ-CT measurement with 3D image processing and statistical analysis, allowed for achieving the following:

- Detecting the inter-cell macro-pores, as follows:
  ○ the ‘real’ (only above ca. 6 µm), due to adaptive geometry and resolution limits of X-ray microtomographic system used.
  ○ the ‘over capillary’ (up to ca. 2000 µm).
  ○ mostly open pores (open porosity ca. 90% in both the W- and S-foam),
  ○ some closed pores (ca. 5000 and 2400).
- Assessing the homogeneity of the pore and strut distributions, with the following conclusions:
  ○ Both the W- and S-foam appeared heterogeneously porous, in terms of the diversity found in the cell and window morphology.
  ○ Several different 3D forms (basic building blocks topologies) could be assigned to the polydisperse cells—irregular dodecahedra (0-12-0), Weaire–Phelan duplex (with two 0-12-0 and six 0-12-2 tetrakaidecahedra per unit cell), Matzke cells (mostly 1-10-2 and negligibly 1-10-3 and 3-7-3-1 14-hedra), and large anisotropic blisters.
  ○ The strut population also resulted in being polydisperse, but seemingly of a homogeneous morphology (no reason was found to reject the independence of the strut 3D form from any blowing gas).
- Finding the processing-to-structure relationship for the PU expanding foam, as follows:
  ○ The relation was found through detecting the morphometric differences between the W- and S-foam, which appeared on macro- and micro-scopic levels.
  ○ The effect of processing on the structure could be explained by highlighting the impact of the interior vapour pressure of the blowing gas mixture (diffused into the growing bubbles) on the initial foam formation. The higher the pressure, the larger sized and the more topologically diversified the cells.
  ○ The environment surrounding the expanding cells (prepolymer) impact is related to viscosity change dynamics and the foam hardening rate. This effect is to be further examined.
- Visualizing the apparent difference in the 3D structures.

## Figures and Tables

**Table 1:** Classification of pores or cells in solids and examples of detection techniques that can resolve given pore size range or topological state.

Pore Type	Detection Techniques
1. Based on size (by IUPAC)	
● Micro-pores (*d* < 2.0 nm):	Scanning tunneling microscopy (STM), transmission electron microscopy (TEM), small-angle X-ray scattering (SAXS), gas adsorption (GA)-α_s_-plot
○ Ultra-micro-pore (*d* < 0.7 nm)	
○ Super-micro-pore (*d* > 0.7 nm)	
● Meso-pore (*d* < 50 nm)	Atomic force microscopy (AFM), GA-Barrett-Joyner-Halenda method (GA-BJH), nano-CT
● Macro-pores (*d* > 50 nm):	Scanning electron microscopy (SEM), mercury intrusion porosimetry (MIP), GA-Density-Functional-Theory method (GA-DFT), polarizing optical microscopy (POM), magnetic resonance imaging (MRI), X-ray computed microtomography (µ-CT)
○ Sub-macro-pore (*d* < 1 µm)	
○ Real macro-pore (*d* < 100 µm)	
○ Over capillary (*d* >1 00 µm)	
2. Based on topology	
● Three-dimensional (3D) geometry	
○ Oval (spherical, anisotropic, channeled)	µ-CT
○ Irregular polyhedron (with polygonal faces)	SEM, TEM
○ Cellular blister (unshaped)	Confocal Microscopy particularly laser confocal scanning microscopy (LCSM)
○ Blowhole (at exterior surface)	
● Openness/closedness	
○ Open pore (transitive)	Gas Pycnometry
○ Mixed pore (dead-ended)	Gas Adsorption
○ Closed pore (latent)	SAXS
3. Based on the foam cells strength	
○ Rigid (hard)	Stress–strain tensometry
○ Flexible (soft)	
4. Based on the origin of formation	
● Intra-particle pores	
○ Intrinsic intra-particle pores	
○ Extrinsic intra-particle pores	
● Inter-particle pores	
● Intra-/inter-grain pores	
● Intra-/inter-cell pores	

**Table 2:** Gas mixture chemical composition and physical properties of the components. S—SUMMER; W—WINTER.

i	W-Foam	S-Foam	Percent Mass *x*_i_ (%)	Molar Mass *M*_i_ (g/mol)	Boiling Point (BP) at 1 atm *T*_i_ (°C)	Saturated Vapor Pressure (SVP) at 20 °C *P*_i_ (kPa)
1	propane	propane	1–10	44.10	−42.1	840
2	dimethyl ether	dimethyl ether	1–10	46.07	−23.6	510
3	isobutane	isobutane	1–10	58.12	−12.0	304
4	1,1-difluoroethane	-	1–10	66.05	−24.7	516

**Table 3:** The polyurethane (PU) foams, panels, and samples specifications.

Parameter (Unit)	Value	
	W-Foam	S-Foam
Panel form in thermal conductivity measurement	cuboid	cuboid
Physical panel dimensions (width; length; height) (mm)	600; 600; 20	600; 600; 20
Sample form in X-ray µ-CT measurement	cylinder	cylinder
Physical sample dimensions (diameter; height) (mm)	20; 20	20; 20
Bulk density of the hardened foam (declared by the manufacturer)—*ρ*_D_ (kg/m^3^)	24 ± 1	22 ± 1
Bulk density of the hardened foam (measured for the sample)—*ρ* (kg/m^3^)	28 ± 2	26 ± 2

**Table 4:** Technical specifications of the scanner.

Scanner Name	SkyScan 1172
Source:	
- Micro Focus X-ray tube type	L7902-20, sealed, air-cooled, Hamamatsu Photonics K.K., Hamamatsu, Japan
- tube current range (µA)	0–250
- tube voltage operational range (kV)	20–100
- X-ray focal spot size (µm)	<5
Detector:	
- X-ray super high transmission (SHT) tube window type	MH110XC-KK-FA, Ximea, Münster, Germany
- detector filters (Al or Al/Cu) (mm)	1 or 0.5/0.040
- camera	12-bit CCD cooled
- scintillator	fiber-optically coupled
- matrix	11Mp (4000 × 2664 pixels)
- detectability (µm)	0.45 (at max resolution)
- image pixel range (µm)	1–25
Sample max size (mm):	
- standard scan	25
- camera offset on	50
- oversized mode on	taller than 25

**Table 5:** The most optimal scanner settings found for data acquisition.

Parameter (Unit)	Value
	W-Foam/S-Foam
X-ray source voltage applied (kV)	33
X-ray source resulting current (µA)	204
X-ray detector binning	1 × 1
Number of rows on camera matrix	2664
Number of columns on camera matrix	4000
Number of lines on camera matrix (random movement amplitude)	40
Detector filter	not activated
Flat field correction	activated
Geometrical correction	activated
Median filtering	activated
Camera offset mode	not activated
Number of connected scans per step (oversized scanning mode)	3
Rotation range (deg)	0–195
Rotational step (deg)	0.2
Rotation sections count (total number of scans)	2925
Number of frames averaged per step	4
Exposure time (ms)	1680
Scanned sample image pixel (square) size (µm)	6.25
Output files type	16-bit TIFF

**Table 6:** The NRecon settings applied in the 3D image reconstruction.

Parameter (Unit)	Value
	W-Foam/S-Foam
Smoothing	0
Misalignment compensation (post-alignment)	−2.0
Ring artifact reduction	1
Beam-hardening correction (%)	10
First section nr (above glue layer)	330/3650
Last section nr (about sample mid-height)	2100/5420
Sections count (number of 2D cross-sectional images)	1771
Region of interest (ROI) (square) size (pixel)	3572
Image pixel (square) size (µm)	6.25
Reconstruction from ROI	activated
Reconstruction angular range (deg)	0–180
2D cross-sectional image size (pixel)	3572 × 3572
3D image voxel dimensions (µm)	6.25 × 6.25 × 6.25
3D cross-sectional image layer dimensions (width; length; thickness) (mm)	22.3; 22.3; 0.00625
3D cross-sectional image stack dimensions (width; length; height) (mm)	22.3; 22.3; 11.1
3D stack files type	8-bit BMP

**Table 7:** The CTAn settings applied in the 3D image analysis.

Parameter (Unit)	Value	
	W-Foam	S-Foam
Resize of the dataset (number of times)	not resized	not resized
Image pixel (square) size (µm)	6.25	6.25
3D image voxel dimensions (µm)	6.25 × 6.25 × 6.25	6.25 × 6.25 × 6.25
ROI (circle) diameter (pixel)	2880	2880
2D cross-sectional image size (pixel)	2884 × 2884	2884 × 2884
First section number	330	3650
Last section number	2100	5420
Sections count	1771	1771
3D cross-sectional image stack dimensions (diameter; height) (mm)	18.0; 11.1	18.0; 11.1
3D stack files type	8-bit BMP	8-bit BMP
Thresholding:		
- lower grey threshold	51	51
- upper grey threshold	255	255
Despeckle:		
- type of item to remove from 3D space	white speckles	white speckles
- max speckle volume (voxels)	38	38
ROI shrink-wrap:		
- space to be shrunk	2D	2D
- stretch over blowholes of a diameter above (pixels)	44	58

**Table 8:** The CTAn output morphometric parameters for W- and S-foam.

Parameter (Unit)	Abbreviation	Value	
		W-Foam	S-Foam
Resize of the dataset (number of times)		not resized	not resized
3D cross-sectional image stack dimensions (diameter; height) (mm)		18.0; 11.1	18.0; 11.1
Total VOI volume (mm^3^)	TV	2825.227	2825.227
Object volume (matrix only) (mm^3^)	Obj.V	326.602	275.411
Volume of closed pores (mm^3^)	Po.V(cl)	0.028	0.011
Volume of open pore space (mm^3^)	Po.V(op)	2498.597	2549.805
Total volume of pore space (mm^3^)	Po.V(tot)	2498.625	2549.816
Number of closed pores	Po.N(cl)	5052	2408
Closed porosity (%)	Po(cl)	0.009	0.004
Open porosity (%)	Po(op)	88.439	90.251
Total porosity (%)	Po(tot)	88.440	90.252
Percent object volume (%)	Obj.V/TV	11.560	9.748
Relative density	*ρ*_r_	0.116	0.097
Total VOI surface (mm^2^)	TS	1171.529	1171.529
Object surface (mm^2^)	Obj.S	35,921.379	29,513.951
Surface of closed pores (mm^2^)	Po.S(cl)	6.131	2.585
Structure separation (mean pore size) (mm)	St.Sp	0.271	0.418
Structure thickness (mean strut thickness) (mm)	St.Th	0.033	0.033

**Table 9:** The extreme gas assumed for calculation.

i	Percent Mass (in Can) *x*_i_ (%)	
	W-Foam	S-Foam
1	1	1
2	1	10
3	1	1
4	10	-

## References

[1] Marques A.C., Dias H., Matos S., Sargaço B., Simoes R., de Schrijver A., Bordado J.C. (2017). Polyurethane one-component formulation optimization for low free isocyanate monomer content. J. Cell. Plast..

[2] Elliott J.A., Windle A.H., Hobdell J.R., Eeckhaut G., Oldman R.J., Ludwig W., Boller E., Cloetens P., Baruchel J. (2002). In-situ deformation of an open-cell flexible polyurethane foam characterised by 3D computed microtomography. J. Mater. Sci..

[3] McDonald S.A., Ravirala N., Withers P.J., Alderson A. (2009). In situ three-dimensional X-ray microtomography of an auxetic foam under tension. Scr. Mater..

[4] Wismans J.G.F., Govaert L.E., van Dommelen J.A.W. (2010). X-ray computed tomography-based modeling of polymeric foams: The effect of finite element model size on the large strain response. J. Polym. Sci. Part B.

[5] Sorrentino L., di Maio E., Iannace S. (2010). Poly(ethylene terephthalate) Foams: Correlation Between the Polymer Properties and the Foaming Process. J. Appl. Polym. Sci..

[6] Shahi P., Behravesh A.H., Rasel S., Rizvi G., Pop-Iliev R. (2017). Morphological Analysis of Foamed HDPE/LLDPE Blends by X-ray Micro-Tomography: Effect of Blending, Mixing Intensity and Foaming Temperature. Cell. Polym..

[7] Luyten J., Mullens S., Cooymans J., de Wilde A.M., Thijs I., Kemps R. (2009). Different Methods to Synthesize Ceramic Foams. J. Eur. Ceram. Soc..

[8] Inayat A., Freund H., Zeiser T., Schwieger W. (2011). Determining the specific surface area of ceramic foams: The tetrakaidecahedra model revisited. Chem. Eng. Sci..

[9] Bracconi M., Ambrosetti M., Maestri M., Groppi G., Tronconi E. (2017). A systematic procedure for the virtual reconstruction of open-cell foams. Chem. Eng. J..

[10] Choudhary A., Pratihar S.K., Agrawal A.K., Behera S.K. (2018). Macroporous SiOC Ceramics with Dense Struts by Positive Sponge Replication Technique. Adv. Eng. Mater..

[11] Salvo L., Suéry M., Marmottant A., Limodin N., Bernard D. (2010). 3D imaging in material science: Application of X-ray tomography. C. R. Phys..

[12] Gaitanaros S., Kyriakides S., Kraynik A.M. (2012). On the crushing response of random open-cell foam. Int. J. Solids Struct..

[13] Waters C., Salih M., Ajinola S. (2015). Porosity comparative analysis of porous copper and OOF modelling. J. Porous Mater.

[14] Fey T., Zierath B., Greil P., Potoczek M. (2015). Microstructural, mechanical and thermal characterization of alumina gel-cast foams manufactured with the use of agarose as gelling agent. J. Porous Mater..

[15] Maruyama B., Spowart J.E., Hooper D.J., Mullens H.M., Druma A.M., Druma C., Alam M.K. (2006). A new technique for obtaining three-dimensional structures in pitch-based carbon foams. Scr. Mater..

[16] Tondi G., Blacher S., Léonard A., Pizzi A., Fierro V., Leban J.M., Celzard A. (2009). X-ray Microtomography Studies of Tannin-Derived Organic and Carbon Foams. Microsc. Microanal..

[17] Inagaki M., Qiu J., Guo Q. (2015). Carbon foam: Preparation and application. Carbon.

[18] Merle J., Sénéchal P., Guerton F., Moonen P., Trinsoutrot P., Birot M., Bouhtoury F.C. (2016). Microstructural characterization of biobased carbon foam by means of X-ray microtomography and compared to conventional techniques. RSC Adv..

[19] Tzvetkov G., Tsyntsarski B., Balashev K., Spassov T. (2016). Microstructural investigations of carbon foams derived from modified coal-tar pitch. Micron.

[20] Maire E., Buffière J.-Y., Salvo L., Blandin J.J., Ludwig W., Létang J.M. (2001). On the Application of X-ray Microtomography in the Field of Materials Science. Adv. Eng. Mater..

[21] Maire E., Withers P.J. (2014). Quantitative X-ray tomography. Int. Mater. Rev..

[22] Nicolaï M.W.B., Verboven P., Swennen R., Roels S., Verstrynge E., Lomov S., Kerckhofs G., van Meerbeek B., Mavridou A.M., Carmignato S., Dewulf W., Leach R. (2018). Applications of CT for Non-destructive Testing and Materials Characterization (Chapter 8). Industrial X-ray Computed Tomography.

[23] Patrick J.W. (1995). Porosity in Carbons: Characterization and Applications.

[24] Inagaki M. (2009). Pores in carbon materials—Importance of their control. New Carbon Mater..

[25] Van Dalen G., Koster M. (2012). 2D & 3D particle size analysis of micro-CT images. Abstract Book.

[26] Sing K.S. (1985). Reporting Physisorption data for gas/solid systems with special reference to the determination of surface area and porosity (Recommendations 1984). Pure Appl. Chem..

[27] Rouquerol J. (1994). Recommendations for the characterization of porous solids (Technical Report). Pure Appl. Chem..

[28] International Union of Pure and Applied Chemistry (1997). Compendium of Chemical Terminology.

[29] Gou H., Nicolae A., Kumar V. (2015). Solid-state poly(methyl methacrylate) (PMMA) nanofoams. Part II: Low-temperature solid state process space using CO2 and the resulting morphologies. Polymer.

[30] Gibson L.J., Ashby M.F., Gibson L.J., Ashby M.F. (1997). The structure of cellular solids (Chapter 2). Cellular Solids: Structure and Properties.

[31] Salvo L., Guilhem M., Suard M., Marmottant A., Dendievel R., Blandin J.J. (2014). Processing and structures of solids foams. C. R. Phys..

[32] Nishikawa K., Yasuda E., Inagaki M., Kaneko K., Endo M., Oya A., Tanabe Y. (2003). Pore Structure Analyses of Carbons by Small-Angle X-ray Scattering (Chapter 11). Carbon Alloys: Novel Concepts to Develop Carbon Science and Technology.

[33] Penttilä P.A., Suuronen J.-P., Kirjoranta S., Peura M., Jouppila K., Tenkanen M., Serimaa R. (2011). X-ray characterization of starch-based solid foams. J. Mater. Sci..

[34] Porada S., Zhao R., van der Wal A., Presser V., Biesheuvel P.M. (2013). Review on the science and technology of water desalination by capacitive deionization. Prog. Mater. Sci..

[35] Mark H.F. (2007). Encyclopedia of Polymer Science and Technology.

[36] Visakh P.M., Nazarenko O. (2017). Nanostructured Polymer Membranes, Volume 1: Processing and Characterization.

[37] Obi B.E. (2017). Polymeric Foams Structure-Property-Performance: A Design Guide.

[38] Coquard R., Baillis D., Quenard D. (2009). Radiative Properties of Expanded Polystyrene Foams. J. Heat Transf..

[39] Baillis D., Coquard R., Randrianalisoa J.H., Dombrovsky L.A., Viskanta R. (2013). Thermal radiation properties of highly porous cellular foams. Spec. Top. Rev. Porous Med..

[40] Raps D., Hossieny N., Park C.B., Altstädt V. (2015). Past and present developments in polymer bead foams and bead foaming technology. Polymer.

[41] Prabhakaran K., Singh P.K., Gokhale N.M., Sharma S.C. (2007). Processing of sucrose to low density carbon foams. J. Mater. Sci..

[42] Ryan S., Hedman T., Christiansen E.L. (2010). Honeycomb vs. foam: Evaluating potential upgrades to ISS module shielding. Acta Astronaut..

[43] Ryan S., Ordonez E., Christiansen E.L. Hypervelocity Impact Performance of Open Cell Foam Core Sandwich Panel Structures. Proceedings of the 11th Hypervelocity Impact Symposium.

[44] Ryan S., Christiansen E.L. (2010). Hypervelocity Impact Testing of Aluminum Foam Core Sandwich Panels.

[45] Zivkovic I., Murk A. (2012). Characterization of open cell SiC foam material. Prog. Electromagn. Res. B.

[46] Yao Y., Chen F., Chen X., Shen Q., Zhang L. (2016). Data of microstructure and mechanical properties of carbon foams derived from sucrose/polyacrylamide hydrogel. Data Brief.

[47] SkyScan N.V. (2005). SkyScan 1172 Desktop X-ray Microtomograph Instruction Manual.

[48] Montminy M.D., Tannenbaum A.R., Macosko C.W. (2004). The 3D structure of real polymer foams. J. Colloid Interface Sci..

[49] Mader K., Mokso R., Raufaste C., Dollet B., Santucci S., Lambert J., Stampanoni M. (2012). Quantitative 3D characterization of cellular materials: Segmentation and morphology of foam. Colloids Surf. A.

[50] Michaeli W., Schrickte L., Berdel K. (2009). Structural Analysis of Polymeric Foam. Abstract Book.

[51] Lang S., Dominietto M., Deyhle H., Müller B. (2001). Imagining of the Tumor Vascularization using µCT. Abstract Book.

[52] Leszczyński B., Wróbel A., Gancarczyk A., Piątek M., Pędryś R. (2014). Morfology of carbon foams. Abstract Book.

[53] Moesen M., Verniers K., Brennan M., Vandenbroeck J. (2015). Morfological analysis of polyurethane foams. Abstract Book.

[54] Moesen M., Verniers K., Wang Y., Brennan M., Vandenbroeck J. (2016). Evaluating heterogeneity of polyurethane bead foams. Abstract Book.

[55] Karimipour-Fard P., Pao W.Y., Rizvi G., Pop-Iliev R. (2018). The Use of Microcomputed Tomography to Evaluate Integral-Skin Cellular Polyolefin Composites. Abstract Book.

[56] Kastner J., Salaberger D., Kickinger R. Structural analysis of polymeric foams by sub-µm X-ray computed tomography. Proceedings of the 10th European Conference on Non-Destructive Testing (ECNDT).

[57] Nacucchi M., de Pascalis F., Scatto M., Capodieci L., Albertoni R. (2016). Structural analysis of advanced polymeric foams by means of high resolution X-ray computed tomography. AIP Conf. Proc..

[58] Chen Y., Das R., Battley M. (2018). An approach for characterizing cellular polymeric foam structures using computed tomography. AIP Conf. Proc..

[59] Yang C.G., Xu L., Chen N. (2007). Thermal expansion of polyurethane foam at low temperature. Energy Convers. Manag..

[60] Feldkamp L.A., Davis L.C., Kress J.W. (1984). Practical cone-beam algorithm. J. Opt. Soc. Am. A.

[61] Bruker microCT (2011). SkyScan NRecon User Guide.

[62] Bruker microCT (2013). Manual for Bruker-microct CT-Analyser Version 1.13.

[63] Bruker microCT (2013). Morphometric Parameters Measured by SkyscanTM CT-Analyser Software.

[64] Bruker microCT (2013). CTvox Quick Start Guide for Software Version 2.4.

[65] Gong L., Jang W.-Y., Kyriakides S. (2005). Compressive response of open-cell foams. Part I: Morphology and elastic properties. Int. J. Solids Struct..

[66] Jang W.-Y., Kraynik A.M., Kyriakides S. (2008). On the microstructure of open-cell foams and its effect on elastic properties. Int. J. Solids Struct..

[67] Buffel B., Desplentere F., Bracke K., Verpoest I. (2014). Modelling open cell-foams based on the Weaire-Phelan unit cell with a minimal surface energy approach. Int. J. Solids Struct..

[68] Gao K., van Dommelen J.A.W., Geers M.G.D. (2016). Microstructure characterization and homogenization of acoustic polyurethane foams: Measurements and simulations. Int. J. Solids Struct..

[69] Kraynik A.M., Reinelt D.A., van Swol F. (2003). Structure of random monodisperse foam. Phys. Rev. E.

[70] Ray S.V. (2011). Microstructural characterization of metal foams: An examination of the applicability of the theoretical models for modeling foams. Mater. Sci. Eng. A.

[71] Thomson S.W. (1887). On the division of space with minimum partitional area. Acta Math..

[72] Kose K. (1996). 3D NMR Imaging of Foam Structures. J. Magn. Res. A.

